# Developing a Mobile App (LYNX) to Support Linkage to HIV/Sexually Transmitted Infection Testing and Pre-Exposure Prophylaxis for Young Men Who Have Sex With Men: Protocol for a Randomized Controlled Trial

**DOI:** 10.2196/10659

**Published:** 2019-01-25

**Authors:** Albert Liu, Kenneth Coleman, Kelly Bojan, Pedro Alonso Serrano, Temitope Oyedele, Amayvis Garcia, Elizabeth Enriquez-Bruce, Patricia Emmanuel, Jeb Jones, Patrick Sullivan, Lisa Hightow-Weidman, Susan Buchbinder, Hyman Scott

**Affiliations:** 1 Bridge HIV San Francisco Department of Public Health San Francisco, CA United States; 2 Department of Medicine University of California, San Francisco San Francisco, CA United States; 3 John H Stroger Jr Hospital Chicago, IL United States; 4 The Ruth M Rothstein CORE Center Chicago, IL United States; 5 Department of Pediatrics University of South Florida, Tampa Tampa, FL United States; 6 Department of Epidemiology Rollins School of Public Health Emory University Atlanta, GA United States; 7 Institute of Global Health and Infectious Diseases University of North Carolina at Chapel Hill Chapel Hill, NC United States

**Keywords:** mobile app, HIV testing, sexually transmitted infections, sexually transmitted diseases, pre-exposure prophylaxis, youth, adolescent, men who have sex with men

## Abstract

**Background:**

Young men who have sex with men (YMSM) in the United States have among the highest incidence of HIV and sexually transmitted infection (STI) and the lowest uptake of HIV and STI testing and pre-exposure prophylaxis (PrEP). Nearly universal mobile phone ownership among youth provides an opportunity to leverage mobile health apps to increase HIV/STI testing and PrEP uptake among YMSM.

**Objective:**

The goals of this project are to develop and refine LYNX, a novel mobile app to support linkage to HIV/STIs testing and PrEP services among YMSM in the United States, and to evaluate the acceptability and feasibility of LYNX in a pilot randomized controlled trial (RCT).

**Methods:**

This research protocol will be conducted in 3 phases: an iterative development phase with a series of 3 focus groups among 20 YMSM to refine the LYNX app; an open technical pilot among 15 YMSM to optimize usability of the app; and then a 6-month pilot RCT among 60 HIV-uninfected YMSM at risk for HIV acquisition. Developed using the Information, Motivation, and Behavioral skills theoretical model, the LYNX app includes an electronic diary to track sexual behaviors (information), a personalized risk score to promote accurate risk perception (information/motivation), testing reminders (motivation/behavioral skills), and access to home-based HIV/STI testing options and geospatial-based HIV/STI testing care sites (behavioral skills). Feasibility and acceptability will be assessed through app analytics of usage patterns and acceptability scales administered via computer-assisted self-interview at 3 and 6 months. We will also evaluate preliminary efficacy by comparing the proportion of YMSM who test at least once during the 6-month pilot and the proportion who successfully link to a PrEP provider in the intervention versus control groups.

**Results:**

Formative work is currently underway. The LYNX pilot RCT will begin enrollment in October 2018, with study results available in 2019.

**Conclusions:**

The LYNX app is one of the first mobile apps designed to increase HIV/STI testing and PrEP uptake among YMSM. As low-perceived risk is a barrier to HIV/STI testing and PrEP use among youth, the personalized risk assessment and interactive sexual diary in LYNX could assist YMSM in better understanding their HIV risk and providing motivation to test for HIV/STIs and initiate PrEP. Coupled with community-based recruitment, this novel mobile app has great potential to reach and engage YMSM not currently involved in care and increase rates of HIV/STI testing and PrEP uptake in this vulnerable population.

**Trial Registration:**

ClinicalTrials.gov NCT03177512; https://clinicaltrials.gov/ct2/show/NCT03177512 (Archived by WebCite at http://www.webcitation.org/73c917wAw)

**International Registered Report Identifier (IRRID):**

PRR1-10.2196/10659

## Introduction

### Background

The HIV epidemic has been worsening among young men who have sex with men (YMSM) in the United States. YMSM aged 13 to 24 years had the greatest increase (26%) in diagnosed HIV infections from 2008 to 2011 [1], and infection rates have remained high through 2014. YMSM accounted for over one quarter of new HIV infections among MSM and over three quarters of new HIV diagnoses among youth aged 13 to 24 years in 2015 [2]. YMSM of color are disproportionately affected by HIV, with black YMSM experiencing the largest increase in new infections during this period. In 2015, black and Latino men who have sex with men (MSM) accounted for 55% and 24% of infections among YMSM, respectively. There is an urgent need for ensuring access to effective HIV prevention approaches in this vulnerable population.

HIV testing is critical for ensuring access to timely treatment and preventing ongoing transmission for HIV-infected YMSM and for linkage to effective preventive tools for those who test HIV negative. Although the Centers for Disease Control and Prevention (CDC) recommends at least yearly HIV testing for MSM [3], in a recent national Web-based survey, only 53% of YMSM reported testing in the past year and 33% had never tested in their lifetime [4]. Furthermore, 44% of HIV-infected youth in the United States were unaware of their diagnosis, compared with 13% of the general population [5]. Reasons for not testing can include factors at the individual, social, and structural levels [6-9] such as not having time to test (11%), low perceived risk (42%), and fear of testing positive/stigma (20%) [10]. Bacterial sexually transmitted infections (STIs) have been identified as potential drivers of HIV infection [11-21]. Despite YMSM having the highest annual STI rate among any age group [22], STI screening rates are low, with less than half of YMSM reporting STI testing in the last year [10,23-30]. Low perceived risk, lack of symptoms, and lower access to health care providers have been identified as barriers to HIV/STI testing [31,32].

Pre-exposure prophylaxis (PrEP) has demonstrated high efficacy, but uptake has been low among YMSM. The iPrEx trial, in which half the participants were aged under 25 years, demonstrated an estimated >90% PrEP efficacy among MSM with detectable drug levels in blood [33,34]. Despite these results, there has been a myriad of challenges to increasing PrEP uptake in the United States, including low awareness, concerns about side effects, low risk perception, and PrEP stigma [35-38]. According to national prescription data, youth aged under 24 years are the least likely to initiate PrEP, with only 9% of PrEP initiations in 2015 occurring in this age group [39]. In a recent national survey, only half of YMSM aged 15 to 24 years had heard of PrEP and 1.7% had ever used PrEP [40]. Demonstration projects also highlight challenges with PrEP uptake. In the US Demo Project of 550 MSM, only 20% were age 25 years or less, and PrEP uptake was lower among younger, nonwhite, and less educated persons. Self-perceived risk was low among those declining PrEP, despite high rates of condomless sex and STIs in this group [41]. In the Adolescent Medicine Trials Network for HIV/AIDS interventions (ATN) 110 study of YMSM aged 18 to 22 years, PrEP uptake was only 16%, and PrEP adherence was lower among black YMSM and declined overall during follow-up, particularly with less frequent visits [42]. Taken together, these data point to deficits in self-perceived risk that may result in low PrEP uptake, especially among YMSM of color, and the importance of engaging youth when offering and delivering PrEP.

Mobile technologies have enormous potential to reach and engage YMSM in HIV prevention [43-47]. Mobile phones have nearly reached saturation among youth, making mobile technology a particularly promising tool to reach this population that has been traditionally hard to reach through clinical services. Smartphone adoption is particularly high among young adults, with approximately 86% of those aged less than 30 years owning a smartphone. Youth are more likely to use their mobile devices for more activities such as downloading mobile apps, internet access, social networking, and accessing health information [48,49], and African American and Latinx individuals are more likely to use their phones for accessing health information and educational content [50]. The expansion of smartphones’ reach has increased the possibilities of dynamic, mobile phone–based HIV prevention interventions.

### Theoretical Framework for Intervention

Using the Information-Motivation-Behavior Skills (IMB) model, we have developed a highly interactive mobile app to promote accurate risk perception and increase HIV/STI testing and linkage to care among YMSM. Key components of the LYNX app will address IMB needs for both increasing HIV/STI testing frequency and PrEP uptake and are shown in Table 1. The sexual health promotion (Sex Pro) tool is an innovative Web-based app [51] that provides a personalized HIV risk score, based on data from several large MSM cohort studies [17,21,52,53]. This score is displayed on a *speedometer* (1-20 scale), with a higher score representing a higher level of protection, and was highly predictive of HIV risk among black MSM in HIV Prevention Trials Network (HPTN) 061, with all HIV infections occurring in individuals with a score below 16 [54]. YMSM found it particularly useful and informative but preferred a mobile app to the Web app format. Sex Pro has been developed into a mobile app, with additional features incorporated, including a sex diary to facilitate accurate data collection; HIV/STI testing information and reminders; access to a home HIV/STI testing kit ordered through a link from the app and delivered to a location of the users’ choosing (eg, home or subject recruitment venue [SRV]); and access to geospatial-based testing site and linkage to HIV care information.

### Aims and Objectives

This study is part of the UNC/Emory Center for Innovative Technology (iTech), which has the overall goal to develop innovative technology–focused interventions addressing the HIV prevention and care continuum for youth [55]. In this protocol, we will first refine the LYNX app through a series of focus groups (FGs) and optimize usability through a small technical pilot. We will then evaluate the acceptability and feasibility of the LYNX app in a pilot randomized controlled trial (RCT) among YMSM at risk for HIV acquisition in the United States. If found to be feasible and acceptable, LYNX will be tested for efficacy in increasing HIV testing and PrEP linkage in a separate efficacy RCT study (COMPARE) as part of the iTech. A costing analysis to determine overall per participant costs for administering the app and cost per HIV test provided and per PrEP initiation will be performed as part of COMPARE.

**Table 1:** LYNX components to increase HIV/STI testing and PrEP uptake, according to the Information-Motivation-Behavior Skills model.

Goal	Information	Motivation	Behavioral skills
Increase HIV/STI^a^ testing	Personalized HIV risk assessment; sexual history diary and partner tracking	Personalized testing reminders; HIV/STI-testing diary and personalized HIV risk score	Home-based HIV/STI-testing options and instructions; geospatial-based testing site and linkage to HIV care information
Increase PrEP^b^ uptake	PrEP educational materials	Testimonials of peers who decided to take PrEP; impact of PrEP on Sex Pro score	Links to youth clinics offering PrEP; app-based tips for insurance/access issues; PrEP navigation through app chat function

^a^STI: sexually transmitted infection.

^b^PrEP: pre-exposure prophylaxis.

## Methods

### Trial Registration, Ethics, Consent, and Institutional Board Approval

This study has been reviewed and approved by the University of North Carolina institutional review board (IRB# 17-0170). Reliance agreements were established for each SRV. A certificate of confidentiality has been obtained from the National Institute of Child Health and Human Development, and a waiver of parental consent/assent will be obtained for participants who are aged 15 to 17 years. The study is also registered on ClinicalTrials.gov (NCT03177512).

### Design

This research protocol will be conducted in 3 phases: an iterative development phase with a series of FGs and in-depth interviews (IDIs) among up to 20 YMSM to refine the LYNX app; an open technical pilot among 15 YMSM to optimize usability of the app; and then a 6-month pilot RCT among 60 HIV-uninfected YMSM at risk for HIV acquisition, in which participants are randomized 2:1 to receive the LYNX app versus standard of care.

Data in the formative phase will include videotaped FGs and IDIs, and data from the technical pilot will include a Web-based qualitative exit interview, a computer-assisted self-interview (CASI), and app analytics (including log-in times, clicks on different app pages, and completion of different app activities). For the pilot RCT, feasibility and acceptability will be assessed through app analytics of usage patterns and acceptability scales administered via CASIs. We will also evaluate preliminary efficacy by comparing the proportion of YMSM who test at least once during the 6-month pilot and the proportion who successfully link to a PrEP provider in the intervention versus control groups. All phases of this study take place in 2 diverse iTech SRV cities: Chicago, IL (study site CORE Center), and Tampa, FL (study site University of South Florida).

### Participants

Eligibility criteria with a “*” are only for participants in the technical pilot and pilot RCT. Eligible participants are cisgender men who (1) are aged 15 to 24 years; (2) have not had an HIV test in the past 6 months* (3 months for the pilot RCT); (3) self-report being HIV-uninfected or HIV status unknown at screening; (4) own an iOS or Android mobile phone and willing and able to download the LYNX app; (5) are able to understand, read, and speak English; (6) are not taking PrEP*; (7) have self-reported evidence of being at risk for HIV acquisition, including at least one of the following in the past 6 months: (a) ≥1 episode of condomless anal sex with an HIV-positive or unknown HIV status male or transfemale partner; (b) anal sex with ≥2 male and/or transfemale partners; (c) exchange of money, gifts, shelter, or drugs for anal sex with a male or transfemale partner; (d) sex with a male or transfemale partner and having had an STI; or (e) if those aged 15 to 18 years who report of any anal sex with a male or transfemale partner; (8) have not received experimental HIV vaccine product with evidence of vaccine-induced seropositivity*; (9) not currently enrolled in another HIV intervention study*; and (10) do not have any health or social condition (eg, cancer requiring frequent hospitalization) that in the judgment of the investigator would make participation unsafe, complicate interpretation of study outcome data, or interfere with achieving study objectives.

To ensure inclusion of youth most impacted by HIV, we will oversample YMSM of color, with a goal of enrolling two-thirds of the cohort YMSM of color and at least one-quarter black YMSM.

### Recruitment

Participants will be recruited through a variety of strategies including Web-based and social media strategies (eg, Craigslist, social networking ads, and gay networking mobile apps); distributing posters, flyers, and palm cards about the study; and direct outreach at local venues frequented by YMSM, including community-based organizations, schools, bars, social clubs, beauty parlors and/or barber shops, sports venues, churches, health fairs, balls, and other community events. Clinic-based recruitment may include reviewing medical charts of existing patients for potential eligibility or referrals from other providers in the clinic.

Potential subjects who are recruited or contact the SRV about the study will be followed up and assessed by trained SRV study staff at participating sites by phone, email, or in person. They will be informed of the nature of the study, the information to be collected, and the evaluations and assessments that are involved. Those who express interest in the study will be required to provide signed informed assent/consent and have eligibility criteria confirmed by research staff before enrolling into the study.

Everyone who is contacted for recruitment, has his medical chart reviewed to assess potential eligibility, is referred to the study by a provider, or is consented for study participation will be referred to a Web-based eligibility screener to assess eligibility. Individuals who do not consent to participate will be asked if they are willing to provide their reason for declining participation. Individuals assessed as ineligible for enrollment will have the reasons for ineligibility recorded.

### Description of App Intervention

The LYNX app has been developed by Apt Mobility (www.aptmobility.com), the technical team who created the original version of the Sex Pro app and has extensive experience creating health-related apps. As shown in Table 1, the LYNX app was developed using the IMB framework to increase HIV/STI testing and PrEP uptake among YMSM. Upon downloading the LYNX app on to a personal device/phone, the user completes an onboarding process. This process includes creating log-in credentials and setting a password to access the password-protected app, entering basic demographic data, customizing user settings (eg, date, time, content of testing and reminders to use Sex Pro, and configuring *rate your partner* categories), and completing the baseline Sex Pro score. In addition to displaying the score, information will be provided on aspects of the participants’ behaviors (eg, number of anal sex partners, condom use, and substance use) that contributed to their score. Participants are then taken to the LYNX landing screen with icons for key functions of the app, including HIV/STI testing and PrEP resources (Information); Sex Pro and earned badges (developed to integrate *gamification* into the app) for completing in-app activities (eg, sexual diary entries and ordering an HIV/STI home testing kit; Motivation); and HIV/STI testing instructions with access to home testing options (Behavioral Skills). All data collected by the app are stored on a secure Web-based cloud environment (Amazon Web Service) that is Health Insurance Portability and Accountability Act (HIPAA)–compliant, with a Business Associates Agreement established for secure data storage. Information is encrypted at rest on the phone and server and during transit to the secure server. Preliminary screenshots of the LYNX app prototype are shown in Multimedia Appendix 1. The LYNX app will be developed for both iOS and Android platforms, the 2 mobile phone platforms that together make up more than 97% of smartphones in the United States [56].

### Formative-Phase Focus Groups

To refine the LYNX app, we will use an iterative development design using FG or individual IDI with 8 to 10 YMSM at each SRV, followed over 3 to 6 months (up to 3 iterations). Each FG or IDI will take 1 to 2 hours. Using a discussion guide, a member of the LYNX study team will demonstrate the app and wireframes (screenshots) of new components and elicit perspectives in content, layout, usability, and functionality. The IMB domains described above will guide the development process. We will also elicit feedback on the home HIV/STI testing kit and instructions and investigate preferences for documentation of clinic or home-based HIV/STI testing (release of medical records, upload test results via secure website and through the app). Participants will be asked to download and test iterative versions of the app for 2 weeks before the next FG, where we will gather feedback on the usability, design, and potential impact of the app. Study staff will use an onboarding document to walk participants through the download procedures and use of the app. FGs may be conducted in person or via videoconferencing.

All FGs and IDIs will be video-recorded for transcription and analysis. The goal of the analysis is to identify barriers and facilitators to app usability. If available, members of the app development team will observe the FGs via video conference in case they need more clarification of specific suggestions for improving the app. Analysis of the data will commence immediately after participants leave, in the form of a discussion of salient themes and suggestions. The team will then prioritize changes to the app for the next iteration to be tested and discussed in the next round of FG discussions. If further transcription is necessary, selected segments of the video and audio interview data will be transcribed.

### Technical Pilot

After revisions to the LYNX app are completed based on the formative phase, we will conduct a 2-month, single-arm, pilot study among 12 to 15 YMSM across 2 iTech SRV cities to optimize the technical performance and functionality of LYNX. YMSM who participated in the formative phase may participate in the technical pilot, as their feedback on app revisions will provide useful insights to the development team. At an in-person enrollment visit (approximately 1.5 hours), YMSM will download the LYNX app and answer a Web-based CASI questionnaire on sociodemographics, use of technology, and risk behaviors. Study staff will use an onboarding document to walk participants through the download procedures and use of the app. Participants will then be encouraged to use all app components over the next 2 months, including ordering and using the HIV/STI test kit at least once during the technical pilot.

Upon completion of the technical pilot, all participants will complete a Web-based exit interview with qualitative-trained study staff to provide feedback on functionality, technical performance, errors and bugs encountered, overall experiences using the app, feasibility and acceptability of methods to confirm HIV testing results (eg, upload results via a secure website), and feedback for further refinement. Web-based interviews (approximately 1 hour) will be conducted via a HIPAA-compliant, video chat application (Zoom) that provides strong security components. We will also assess youth satisfaction with the app using the system usability scale (SUS), a validated assessment tool assessing various domains of the app with demonstrated high internal consistency across a number of studies [57]. Participants will be emailed a link to complete a CASI to assess these measures. Each CASI will take approximately 1 hour to complete. All exit interviews will be audio- and video-recorded and transcribed for analysis by the iTech Analytic Core (AC).

### Pilot Randomized Controlled Trial

After the LYNX app is refined and optimized through the findings from the formative work and technical pilot, we will evaluate the feasibility, acceptability, and preliminary impact of LYNX through a pilot RCT with 60 HIV-uninfected YMSM at risk for HIV acquisition.

#### Randomization

Participants who express interest in using LYNX, meet eligibility criteria, provide informed consent, and complete a baseline assessment will be eligible for enrollment and randomization. The enrollment visit will take approximately 1.5 hours. After successful enrollment into the study, subjects will be randomized 2:1 into either the LYNX intervention arm (N=40) or control arm (N=20). The 2:1 allocation will allow us to efficiently gather additional data on app utilization. Randomization will be stratified by age (15 to 18 years and 19 to 24 years) and site and based on a pregenerated list created by the lead iTech AC statistician, with random blocks of size 3 or 6 [58]. After assent/consent and baseline survey completion, the participant will be randomized (assigned to the next available random allocation).

#### LYNX Intervention Condition

Individuals who receive LYNX will be given brief instructions on the purpose of the app, how to access it, and an overview of how to use it. Participants will be encouraged to explore all components of the app and use it routinely.

#### Standard of Care Condition

Following screening, participants in both conditions will receive standard of care prevention material consisting of provision of information regarding recommendations for HIV testing and referrals to local HIV testing sites and prevention services.

#### Intervention

Study staff will assist participants in downloading the LYNX mobile app, provide instruction on its use, and help set up reminders to input sex diary entries. To restrict access to the LYNX app to intervention arm participants only, they will be provided a single-use registration code that will need to be entered to gain access to the app. Study staff will use an onboarding document to walk participants through the download procedures and use of the app. These reminders will be personalized by the user for day, time, and message content at the first visit and can subsequently be updated by participants if desired. Participants will be encouraged to explore and use other components of the app (including the Sex Pro score, PrEP videos, bidirectional chat function with study staff, and geolocation features). Users will receive quarterly HIV/STI testing reminders using the mobile device notification feature. For confidentiality purposes, reminder notifications are nonspecific, but inside the app, the participant is linked to a customizable reminder. Reminders include 2 options for testing: (1) the ability to order a home HIV/STI testing kit to be mailed to a location of their choosing (eg, home or the SRV) free of charge or (2) a geo-located map of the closest HIV/STI testing sites. For participants who test HIV-positive during the study, information about next steps for linkage to care is included in the testing section of the app, including a phone number for an on-call clinician available 24 hours a day. In addition, there is a chat function in which participants can contact LYNX staff for support and assistance with linkage to care. Any participant who enters a positive HIV test result into the app will be contacted by the study team and provided supportive counseling and referral to treatment services.

#### Control Arm

Participants randomized to the control condition will be instructed to access HIV/STI testing at existing sites in the community. They will be provided with a list of these testing sites, along with an informational brochure about PrEP. All participants will be provided access to Sex Pro (risk assessment tool that is part of LYNX and will be made available on the Web) after completion of the study.

#### Follow-Up Visits

All enrolled participants will be followed for 24 weeks. After enrollment, all follow-up visits will occur on the Web, with SMS reminders, email, and phone follow-up conducted as needed to ensure completion of study procedures. Participants will complete a Web-based CASI at 12 and 24 weeks (each approximately 1 hour) and receive a stipend for completion of procedures at each visit. Visit windows will be 14 days before or after the target date.

At the 3-month visit, up to 20 participants randomized to the LYNX arm will be selected for participation in a 1.5-hour exit IDI. The purpose of the exit interviews is to elicit additional feedback on their experiences using the app, any technical difficulties encountered, and how the app could be further improved. Participants will be selected for interview using purposive sampling based on level of engagement with the app, whether participants completed HIV/STI testing and/or initiated PrEP during the study, and to achieve diversity based on sociodemographics (eg, age and race/ethnicity). Additionally, any participant who has a positive HIV test during the study will be offered an interview. By purposively sampling certain participants for the exit interviews, the goal is to select *information-rich* cases from which one can learn a great deal about issues of central importance to the purpose of the research [59]. All interviews will be conducted via Zoom and audio-recorded for transcription and analysis.

### Outcomes

Primary and secondary outcomes and moderating variables for the pilot RCT are shown in Table 2.

The primary acceptability outcome will be measured by the SUS, a 10-item, 5-point Likert scale giving an overall view of usability. The SUS is technology-independent and provides a global measure of system satisfaction and subscales of usability and learnability [57]. A score of >50 (out of 100) indicates the app is acceptable [74]. We will also assess interest in future use of LYNX at study completion. For feasibility, we will assess frequency of log-ins and use of various components of LYNX, based on app analytics. If >60% of participants open the app at least once after the initial enrollment visit, it will be considered feasible. We will also assess the proportion of YMSM who complete the personalized risk assessment, a key component of the app, and the number of HIV/STI home testing kits requested and completed. As a secondary outcome, we will evaluate the preliminary efficacy of LYNX in increasing HIV/STI testing and PrEP care linkage. For HIV testing, we will evaluate the proportion who complete at least one HIV test during the 6 months of follow-up. Self-reported HIV/STI testing via CASI will be confirmed by methods that are finalized during the formative phase (eg, medical record review and upload test results via secure website). For PrEP linkage, we will evaluate (1) the proportion of participants reporting interest in PrEP uptake during follow-up using the question “How interested are you in taking PrEP?” with response options “not at all interested, a little interested, somewhat interested, very interested, and extremely interested” compared with baseline; (2) the proportion making and attending a clinic appointment for PrEP evaluation; and (3) the proportion who receive a prescription for PrEP and the proportion who pick up PrEP medication from the pharmacy. We will request participants to sign medical release forms for the release of their HIV/STI testing results and records regarding PrEP initiation. We will also measure sexual behaviors at each quarterly follow-up visit, including numbers of partners and types of sexual behaviors, by HIV serostatus and position (insertive and receptive) in the past 3 months. Finally, we will assess IMB model constructs including HIV/STI testing and PrEP knowledge, attitudes, motivations, and behavioral skills related to HIV/STI testing and PrEP uptake. Given the importance of moderating contextual factors that may influence uptake of prevention strategies in YMSM, we will use the eco-social theoretical model which links individual, social, and structural factors (ie, socioeconomic position, social networks, and stigma) across a hierarchical framework to explore these factors as they relate to HIV/STI testing and PrEP uptake among YMSM [6-9]. Process measures including recruitment, consent, dropout, and missed visit rates will also be assessed.

### Statistical Analysis

Response rates to follow-up surveys will be tabulated by recruitment venue and respondent characteristics to help understand potential sources of bias. We will characterize the study population using descriptive statistics and compare the intervention and control groups at baseline using *t* test, Wilcoxon test, and chi-square test. The primary outcomes of the pilot RCT will be acceptability and feasibility of the app. Point estimates for mean SUS score ≥50 and for proportion accessing the app >0.60 will be considered the minimum criteria for acceptability and feasibility, consistent with industry standards [57]. Descriptive statistics will be used to evaluate app analytics, including number of log-in attempts, cumulative time spent using the app, mean session duration, and frequency and duration of use of different components of the app. A secondary feasibility outcome will be achieved if ≥50% of participants who open the app complete their personalized risk score at least once.

The secondary outcomes of preliminary efficacy of the LYNX app to increase HIV/STI testing (any testing over follow-up) and PrEP uptake (as described above) will be evaluated using unadjusted risk ratios for each outcome. If there is evidence of divergence from balance in measured baseline covariates (ie, failure of randomization), post hoc analyses using Poisson regression with robust SEs [75] will be used to estimate adjusted risk ratios. Outcome variables will represent any HIV testing and any PrEP uptake over the follow-up period. Separate models will be estimated for each outcome.

With 60 participants randomized 2:1 to intervention:control and 10% to 20% attrition, we will have 80% power to detect 37% to 42% point increases in HIV testing and PrEP uptake, depending on rates in controls. The lack of precision and large minimum detectable effects, typical of pilot studies, will entail careful interpretation of study results in the light of overall patterns, plausibility, and findings from other mobile health (mHealth) studies.

### Incentives

Participants receive incentives consistent with local standards for completing each study visit. This includes the US $50 to US $60 for the baseline visit and US $25 to US $30 for the 3- and 6-month follow-up visits.

**Table 2:** Outcomes and measures for LYNX.

Domain			Data source	Description/scale
**Primary outcomes**				
	Acceptability		CASI^a^	System Usability Scale [57], Intervention Acceptability Scale [61], and acceptability of app components
	Feasibility		App analytics	Frequency of app log-ins and use of different components of LYNX
**Secondary outcomes**				
	HIV testing frequency		CASI, EP^b^, MR^c^	Number of HIV tests during study
	STI^d^ testing frequency		CASI, EP, MR	Number of STI tests during study
	HIV testing knowledge, attitudes, and behaviors		CASI	National HIV Behavioral Surveillance men who have sex with men-4 (NHBS-MSM-4)
	PrEP^e^ knowledge and attitudes; PrEP willingness		CASI	PrEP awareness and willingness scales
	PrEP linkage		CASI, MR	HIV-negative cascade measures
	Social impacts		CASI	Social benefits and harms of using app
**Model constructs**				
	Information-Motivation-Behavioral Skills (IMB)		CASI	Adapted scales based on content of app [62,63]
**Covariates (based on eco-social model)[6-9]**				
	**Individual**			
		Demographics, socioeconomic position	CASI	Age, race/ethnicity, gender identity, sexual identity, student status, education, income, family structure, employment, insurance status, and food insecurity
		Sexual behavior (number of sex partners, condom use, partner selection)	CASI	Numbers and types of partners, HIV-status of partners, sexual position, and condom use
		Drug use behavior (ie, alcohol, cocaine, meth)	CASI	Alcohol, Smoking and Substance Involvement Screening Test (ASSIST) [64] and Alcohol Use Disorders Identification Test (AUDIT-C) [65]
		Self-efficacy	CASI	HIV testing [66], PrEP use [67], condom use [68]
		Mental health (depression, anxiety)	CASI	Generalized anxiety disorder 7-item scale and patient health questionnaire
		Perceived risk of HIV infection	CASI	Perceived risk of HIV scale [69]
		Trauma and abuse	CASI	Startle, Physiological Arousal, Anger and Numbness (SPAN) [70]
	**Social/sexual network**			
		Social support	CASI	Patient-Reported Outcomes Measurement Information System (PROMIS) [71]
		Peer norms for condom use	CASI	
	**Structural**			
		SRV^f^/city	CRF^g^	Geographic location of study participant
		Access to health care	CASI	Barriers, frequency of seeing a provider, locations, and comfortability in discussing sex with provider
		Incarceration history	CASI	Ever and recent history
		Structural discrimination	CASI	Everyday discrimination scale [72], racism, sexual minority stress [73], and medical mistrust
		Stigma	CASI	PrEP-related and sexuality-related
**Other covariates**				
	Mobile phone and technology use		CASI	Device types, operating system, and phone plan, sharing of devices, internet use and frequency, and use of social networking sites
	Mobile app use over study period		App analytics	Login attempts, HIV testing and PrEP use, proportion complete HIV-testing plan, and proportion requesting HIV/STI home test kits

^a^CASI: computer-assisted self-interview.

^b^EP: electronic picture.

^c^MR: medical record confirmation.

^d^STI: sexually transmitted infection.

^e^PrEP: pre-exposure prophylaxis.

^f^SRV: subject recruitment venue.

^g^CRF: case report form.

## Results

This pilot RCT will begin enrolling in October 2018, and study results will be available in 2019.

## Discussion

The LYNX pilot RCT will evaluate the feasibility and acceptability of one of the first mobile apps designed to increase HIV testing and PrEP uptake among YMSM. A recent review of 285 HIV mobile apps revealed that only 8% specifically addressed MSM and none dealt with PrEP [76]. Furthermore, most were developed by nonacademic, nonpublic health developers, which may be less credible than apps created by university or health department sponsors [77]. LYNX was developed by a multidisciplinary team of public health researchers, HIV physicians, behavioral specialists, and app developers, with input from YMSM at all stages of development. Although most prior mHealth prevention interventions have focused on behavioral risk reduction alone, LYNX will incorporate components to increase HIV/STI testing and linkage to treatment and prevention services, including PrEP, and will be specifically tailored for use in YMSM.

The LYNX app will use a personalized risk assessment to assist YMSM in evaluating their risk of HIV. Internet-based personalized decision support tools have been used successfully to assist users with behavior changes in several diseases including heavy alcohol use, hyperlipidemia, and obesity [78-85]. Currently, no mHealth interventions integrate personalized HIV risk assessment, appeal to YMSM, and incorporate HIV/STI testing and resources to increase PrEP uptake [86-88]. A highly engaging risk assessment tool could assist YMSM better understand their HIV risk, provide motivation to test for HIV/STIs, and help determine when to take PrEP.

Challenges in developing mHealth interventions have been described previously and include coordination and communication with the technology developer; understanding the needs and preferences of the target population, vendors, and researchers having differing values and priorities; and extra time needed for testing and debugging the intervention [89]. To address these potential challenges in the development and testing of the LYNX app, we have established weekly calls with Apt Mobility to discuss development priorities and provide feedback on app development; established a Web-based spreadsheet to track all requests for app builds and modifications; and assigned a study coordinator with extensive experience in developing mHealth apps to manage this project. In addition, as described in this protocol, we have included extensive formative work to elicit feedback from diverse YMSM regarding their preferences and needs for content and features. Finally, we have worked with the iTech core to build realistic timelines, factoring in time required for app testing, debugging, and revisions.

In summary, a technology-based HIV prevention app has great potential in scaling up HIV/STI testing and PrEP use among YMSM. Modeling studies in MSM suggest that substantial coverage of HIV prevention services is needed to reduce population-level incidence [90], yet uptake of these services has been limited in youth. With the high penetration of smartphones among youth, LYNX has great potential to reach large numbers of high-risk YMSM who may not access medical care or traditional testing sites, at marginal incremental costs. If found to be feasible and acceptable in this pilot study, the LYNX app will be evaluated in a head-to-head comparison with the MyChoices app (Biello et al, *ATN 141,* submitted to this issue) in the COMPARE RCT. If effective, these apps could facilitate nationwide scale-up of HIV/STI testing and PrEP use among YMSM.

## Appendix

Multimedia Appendix 1 Screenshots of the LYNX app.

## Abbreviations

ATN: Adolescent Medicine Trials Network
CASI: computer-assisted self-interview
CRF: case report form
EP: electronic picture
FG: focus group
IDI: in-depth interview
IMB: Information-Motivation-Behavioral Skills
mHealth: mobile health
MR: medical record confirmation
MSM: men who have sex with men
PrEP: pre-exposure prophylaxis
RCT: randomized controlled trial
Sex Pro: sexual health promotion
SRV: subject recruitment venue
STI: sexually transmitted infection
SUS: System Usability Scale
YMSM: young men who have sex with men
