# Supplementary material for: Developing a Mobile App (LYNX) to Support Linkage to HIV/Sexually Transmitted Infection Testing and Pre-Exposure Prophylaxis for Young Men Who Have Sex With Men: Protocol for a Randomized Controlled Trial
Source: JMIR Res Protoc. 2019 Jan 25;8(1):e10659. doi: 10.2196/10659 (PMC6367663; doi:10.2196/10659)
Supplement: Multimedia Appendix 1 [file resprot_v8i1e10659_app1.pdf]

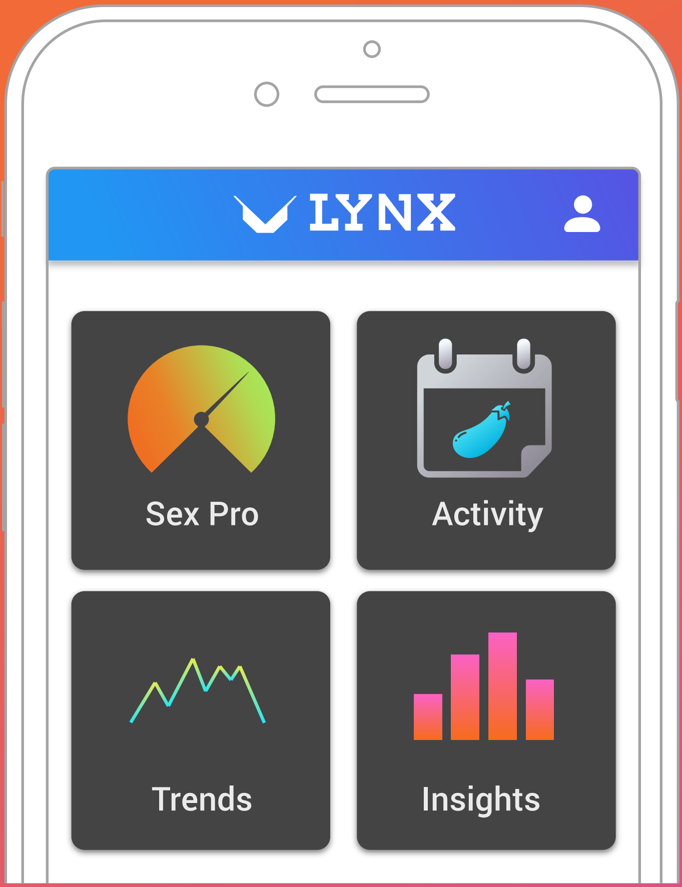

Welcome to Lynx!

Lynx is all about blending pleasure with HIV prevention. It'll help you track, check, and protect so you can focus on the pleasure of sex.

NEXT

YOUR SCORE WILL UPDATE IN **90 DAYS**

Baseline Score

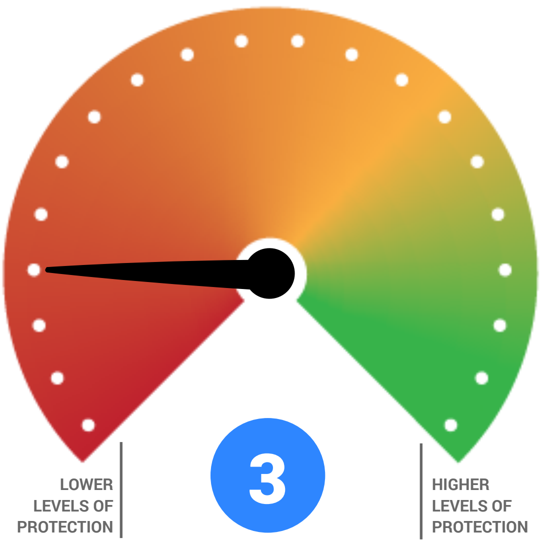

Want to get into the green? PrEP is a great way to make it happen & protect your sexual health. Hit us up, we can help.

Why did I get this score?

You had anal sex with [#] people

You used condoms [%] of the time as a top

You used condoms [%] of the time as a bottom

You used meth in the last 3 months

You used cocaine or crack in the last 3 months

You used poppers in the last 3 months

You had an STD in the last three months

You were drinking heavily in the last three months

*** Learn why these matter**

GOT IT!

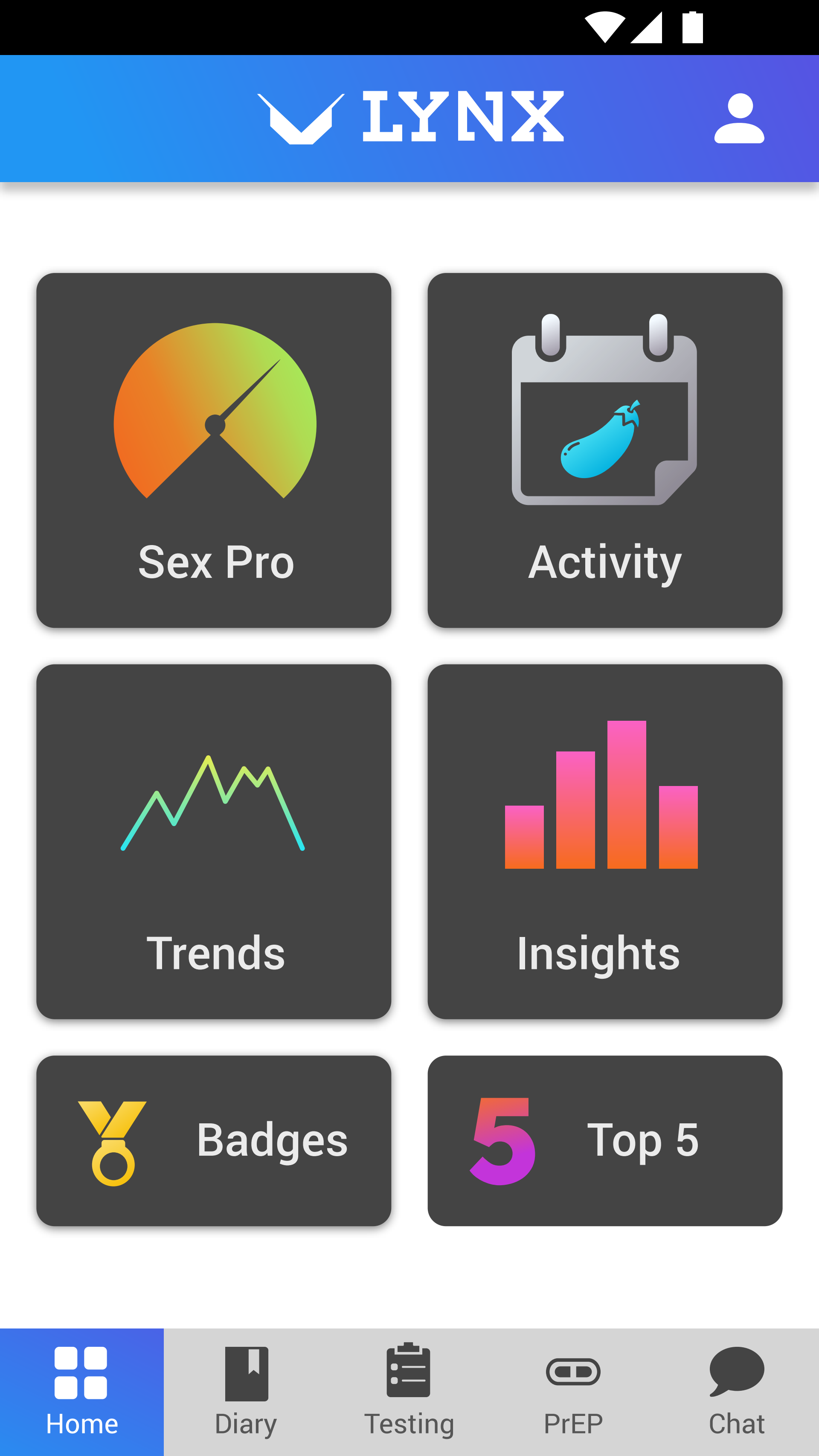

LYNX
Sex Pro
Activity
Trends
Insights
Badges
5
Top 5
Home
Diary
Testing
PrEP
Chat

Your Sex Trends

For [xx] encounters with [xx] partners:

60% — *Exclusively a bottom*

25% — *Exclusively a top*

25% — *Versatile*

15% — *Used condom during sex*

15% — *Used condom as a bottom*

15% — *Used condom as a top*

25% — *Your partner came in you*

15% — *You came in your partner*

25% — *You swallowed*

15% — *You had sex drunk and/or high*

---

3 — *# days since last HIV test*

2 — *# days since last STD test*

1 — *# of partners you topped with*

2 — *# of partners you bottomed with*

1 — *# of 5 star encounters*

Your Partner Insights

For [xx] encounters with [xx] partners:

Your Partners

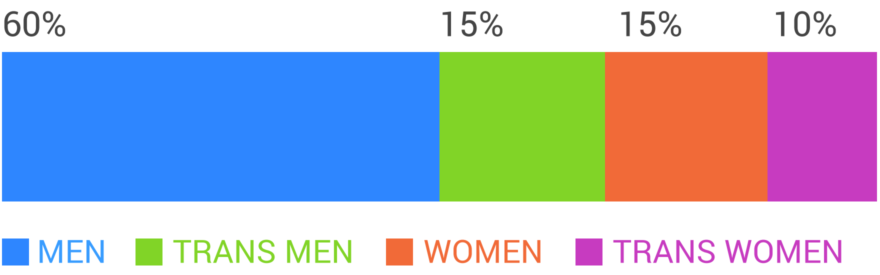

Partner types

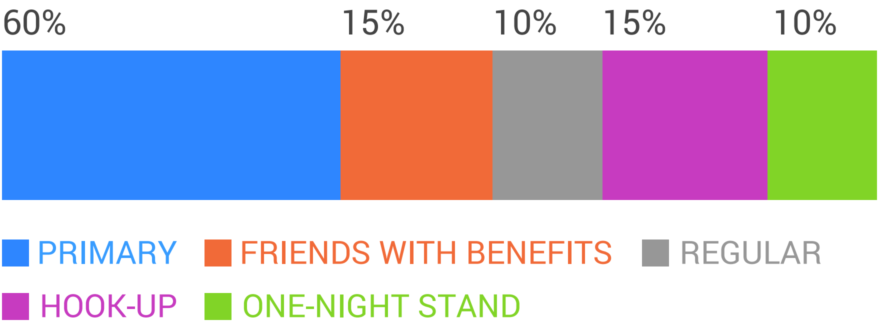

Partner HIV Status

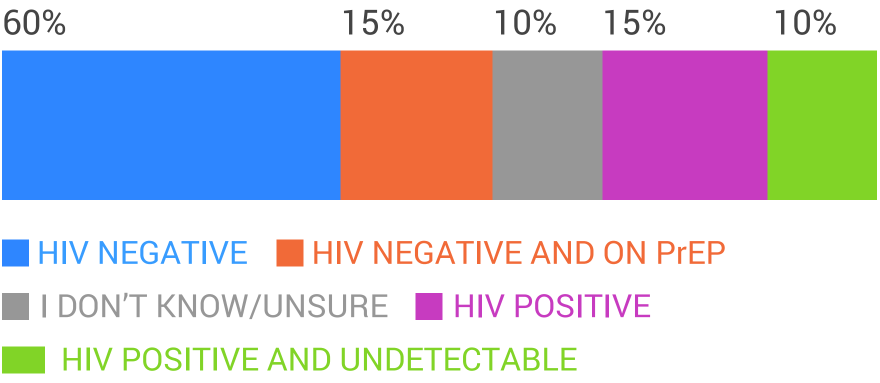

3

# of HIV postive partners

2

# of HIV negative partners

1

# of unknown status partners

0

# of 5 star partners

1

# of repeat partners

0

# of partners on PrEP

1

# of undetectable partners

New Encounter

Who did you have sex with?

ADD NEW PARTNER

William Shakespeare

William Shakespeare

NEXT

New Partner

MICHAEL

Rate your partner

Overall

Chemistry

Personality

Face

Add choice

Add choice

Add choice

NEXT

LYNX

HISTORY | TEST KIT | LOCATIONS

ADD NEW HIV TEST

ADD NEW STD TEST

12/30/17	Chlamydia	Positive	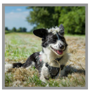
12/30/17	Syphilis	Negative	
12/30/17	HIV	Negative	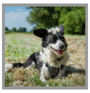

Home | Diary | Testing | PrEP | Chat

Add New STD Test

When was your most recent STD test?

07/15/2017

Were any of your STD tests postive?

Gonorrhea

- (●) No
- ( ) Yes
- ( ) I didn't test

Syphilis

- (●) No
- ( ) Yes
- ( ) I didn't test

Chlamydia

- (●) No
- ( ) Yes
- ( ) I didn't test

SAVE

LYNX

HISTORY | TEST KIT | LOCATIONS

Enter your city or zip code

Filters

- PrEP
- HIV Testing
- STI Testing

10 miles

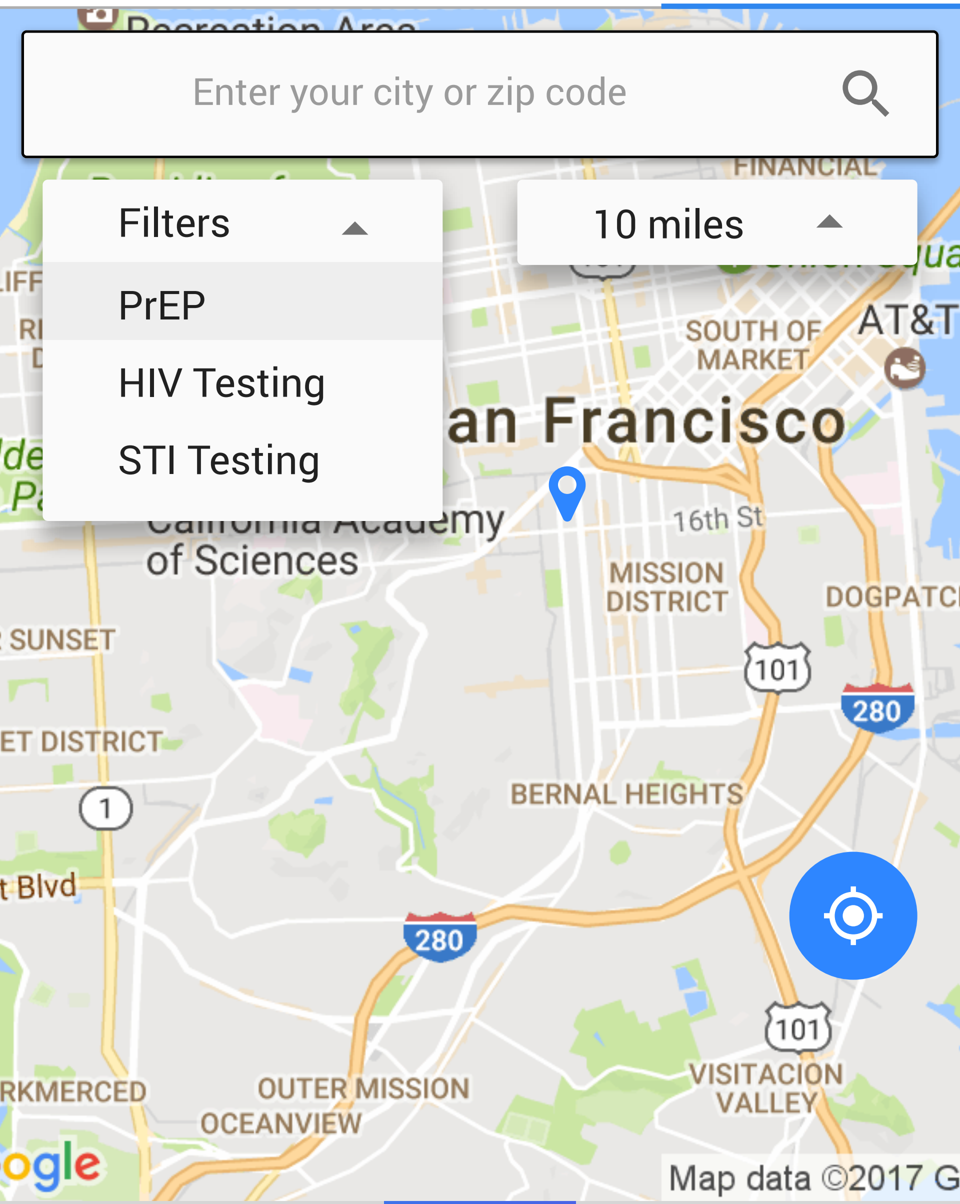

Home | Diary | Testing | PrEP | Chat

LYNX

NEED US NOW?

You should have received your test result by now. Let me look and see what I can find.

Lynx Study, Sep 11, 3:38 PM

Thanks so much. Can you also look and see if my new STD kit has been sent to me already?

You, Sep 11, 3:42 PM

Your STD kit is on the way. I'm still checking on your test result.

Lynx Study, Sep 11, 3:38 PM

Message

Home Diary Testing PrEP Chat

High Five

Entered first encounter

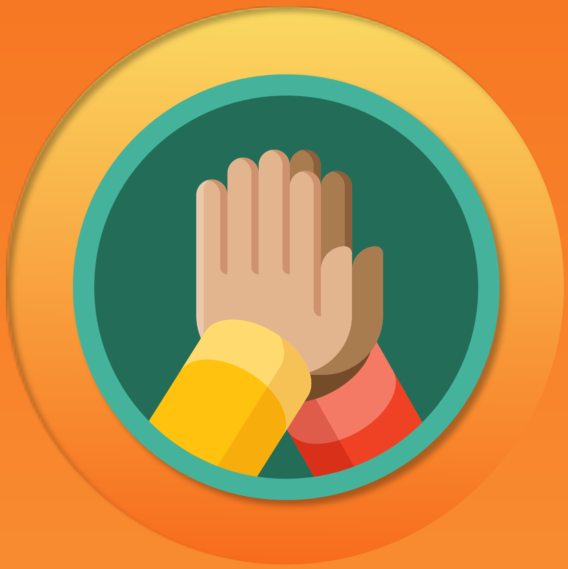

Yay! Shout out to the first timers.
Congrats on completing Entry #1.

Earned 1 time

PrEP'd

Started PrEP

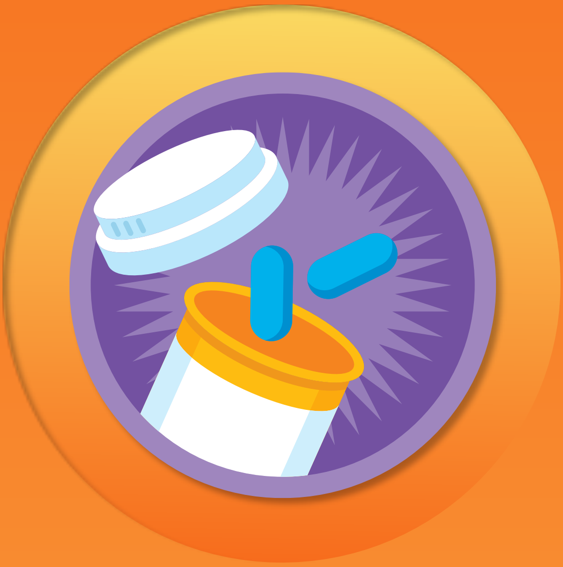

A daily commitment to take PrEP is
a commitment to yourself.
Good lookin' out.

Earned 1 time

Golden Penis

100% condom use as a top in a month

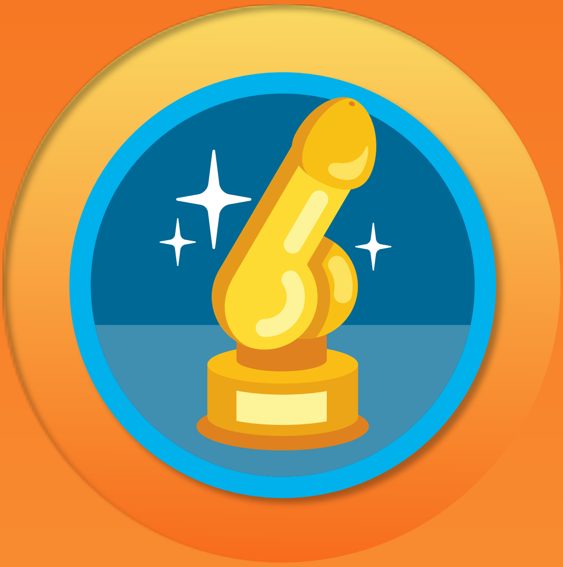

You won the big one for
keeping it wrapped!

Earned 1 time
